# Perception of telemedicine among medical practitioners in Malaysia during COVID-19

**DOI:** 10.25122/jml-2020-0119

**Published:** 2021

**Authors:** How Kit Thong, Danny Kit Chung Wong, Hardip Singh Gendeh, Lokman Saim, Primuharsa Putra Bin Sabir Husin Athar, Aminuddin Saim

**Affiliations:** 1. Department of Otorhinolaryngology, Head and Neck Surgery Faculty of Medicine, KPJ Healthcare University College, Negeri Sembilan, Malaysia; 2. Department of Otorhinolaryngology, Head and Neck Surgery, Faculty of Medicine, Universiti Kebangsaan Malaysia, Kuala Lumpur, Malaysia; 3. Department of Otorhinolaryngology, Head and Neck Surgery, KPJ Tawakal Specialist Hospital, Kuala Lumpur, Malaysia; 4. Department of Otorhinolaryngology, Head and Neck Surgery, KPJ Seremban Specialist Hospital, Negeri Sembilan, Malaysia; 5. Department of Otorhinolaryngology, Head and Neck Surgery, KPJ Ampang Puteri Specialist Hospital, Selangor, Malaysia

**Keywords:** telemedicine, telehealth, COVID-19, coronavirus

## Abstract

The novel Coronavirus Disease 2019 (COVID-19) has brought unprecedented changes in the way conventional health care is delivered. This study examined if clinicians’ perceptions regarding telemedicine and its barriers to implementation in Malaysia have changed during this pandemic. A cross-sectional survey was conducted among Malaysian medical doctors of various specialties in four urban healthcare facilities between June 2020 and July 2020. A total of 146 (41.7%) out of 350 responses were obtained. 62% of doctors reported a reduction greater than 50% in outpatient visits during the COVID-19 pandemic. The majority of doctors either found telemedicine useful in situations similar to COVID-19 (34.2%) or that it is essential to their daily practice (42.5%). However, only 22% reported using telemedicine for consultation during the COVID-19 pandemic. 74% of doctors felt that telemedicine would only benefit up to 30% of their patient population. Significantly more female doctors (80%) felt that telemedicine would benefit their patients compared to male doctors (45.8%) (P=0.03). Physicians (51.3%) were more inclined to adopt telemedicine in comparison to surgeons (32.4%) (P=0.03). The majority cited medico-legal issues and consent (80.6%), billing and charges (66.7%) and insurance reimbursement (62.5%), technical difficulties (62.5%) as their barrier to the adoption of telemedicine. Female doctors and physicians were more willing to adopt telemedicine when compared to male doctors and surgeons. Although the COVID-19 pandemic appeared to improve the perception, significant barriers should be resolved before many can incorporate it into their practice.

## Introduction

The novel Coronavirus Disease 2019 (COVID-19) first emerged as a series of severe pneumonia of unknown etiology in Wuhan, Hubei Province of China on 31 December 2019. The causative agent, known as Severe Acute Respiratory Syndrome Corona Virus 2 (SARS-CoV-2), was isolated on 7 January 2020. Since then, many countries have enforced social isolation or social distancing through “lockdown” to reduce the spread of disease. In Malaysia, this was termed the “Movement Control order” (MCO) implemented on 18 March 2020 and relaxed on 10 June 2020 [1].

During MCO, Malaysian hospitals had to adapt and comply with new recommendations, resulting in unprecedented changes in the way conventional health care was delivered. These changes involved a suspension of elective surgical procedures, rescheduling non-essential outpatient appointments, and a reduction or redistribution in hospital staff for COVID-19 and hybrid COVID-19 hospitals. Doctors who previously relied on face-to-face encounters were forced to find alternative ways to continue providing care to their patients, thereby creating a renewed interest in telemedicine [2, 3].

This was seen in many countries in “lockdown” such as the United Kingdom (UK), United States of America (USA), China, and Australia, where healthcare providers turned to the use of telemedicine, particularly video consultations, as it would limit physical encounters and reduce the risk of infection [2, 3]. As the pandemic worsened, similar efforts to accelerate telemedicine were seen in Vietnam. Hanoi Medical University Hospital introduced a “digital hospital” project that aimed to encourage telemedicine as a way for both doctor-to-patient and doctor-to-doctor communication. Preliminary results of the effort have revealed the promising role of such implementation to enhance the quality of healthcare service delivery in the digital era [4].

Telemedicine is the practice of medicine using audio, video and data communications, and it can be divided into three categories. The first is patient monitoring at home. The second category consists of real-time interactive or live interactive online applications. These include teleconsultation, videoconferencing, telesurgery, and similar applications. The third category includes store-and-forward applications that use non-interactive technology [5].

During the COVID-19 pandemic, telemedicine has been shown to be beneficial not only with pandemic-related queries but in other disciplines of medicine such as mental health, otorhinolaryngology, urology, ophthalmology, orthopedic surgery and oncology [6–11]. In New York, USA, a reliable health system reported a significant increase in telemedicine usage from 369.1 to 866.8 daily (a 135% increase) in urgent care cases and from 94.7 to 4209.3 per day (a 4345% increase) in non-urgent care cases [12]. In the UK, general practitioners have noted 12,000 video consultations per day, which is significantly higher than the previous 300 consultations per month [13].

The increased reliance on telemedicine has inspired several professional organizations, including the European Association of Urology, to create guidelines for the implementation of telemedicine in routine urological practice. In the guidelines, 14 recommendations were introduced to ensure best practices for telemedicine in urology [14]. Optimal implementation will uphold the quality of care received by patients, and the outcomes of patients are of the highest standard.

This study examined if there has been any change in the perceptions of doctors regarding telemedicine, willingness to adopt it, and its barriers to implementation here in Malaysia during this COVID-19 pandemic. The focus was primarily on the second category of telemedicine (i.e., teleconsultation, videoconferencing, or text chat through online applications).

## Material and Methods

### Objectives

The objectives of the study were to:

• Investigate the perception of telemedicine before and during the COVID-19 pandemic;
• Determine the willingness of doctors to adopt telemedicine;
• Explore the barriers to the implementation of telemedicine in Malaysian healthcare.

### Study design and ethics approval

This was a cross-sectional survey in which a self-administered online questionnaire entitled “Is telemedicine relevant in your practice?” was conducted among Malaysian medical practitioners between June 2020 and July 2020. Before the commencement of the study, full ethical approval was obtained from the Research and Innovation Centre of KPJ Healthcare University College. (Approval no: KPJUC/RMC/EC/2020/283)

### Study population

The questionnaire was distributed to 350 doctors in four private hospitals covering four states, namely KPJ Ampang Puteri Specialist Hospital, KPJ Seremban Specialist Hospital, KPJ Damansara Specialist Hospital and KPJ Penang Specialist Hospital. The participants were from private urban healthcare centers involving consultants from various specialties. Nurses and other allied healthcare professionals were excluded.

### Preparation of questionnaire

The survey was an online questionnaire distributed via e-mail through a Google document format. It was a 16-question self-administered survey.

It was designed and modified based on previously published research articles [15]. A group of two-member experts with experience in the field of telemedicine evaluated the modified questionnaire. The questions were either dichotomous (yes/no) responses or multiple-choice questions.

The questionnaire consists of five domains (Appendix 1):

1. Demographic characteristics (Questions 1–5);
2. Impact of COVID-19 on the healthcare economy (Questions 6–7);
3. Intention to use telemedicine (Questions 8–10);
4. Knowledge and awareness about telemedicine (Questions 11–12);
5. Perceived difficulties in implementing telemedicine (Question 13);
6. Organization readiness (Question 14–16).

### Statistical analysis

Statistical analyses will be performed using the Statistical Package for the Social Sciences (SPSS) software (Version 24.0, IBM Corp., Armonk, NY). Descriptive statistics were used in the form of frequencies and percentages for categorical variables. The Chi-squared test was used to measure the association between some of the variables in the study. A p-value ≤0.05 was considered statistically significant.

## Results

There were 146 respondents out of the 350 invitations sent (41.7%). The majority of the respondents were male (65.75%), with more than 20 years of experience working as a healthcare professional (64.4%). The source of knowledge and awareness regarding telemedicine came mainly from social media and news (69.9%). There were still 4% of the respondents who reported no knowledge of telemedicine at all (Table 1).

**Table 1.** Demographic profile of doctors who responded to the questionnaire.

**Characteristics**	**N (N=146)**	**Percentage**
**Gender**		
**Male**	96	65.75
**Female**	50	34.25
**Department of Practice**		
**ENT**	28	19.2
**Emergency Medicine**	18	12.3
**Internal Medicine**	18	12.3
**Family Medicine**	12	8.2
**Pediatrics**	12	8.2
**Obstetrics and Gynecology**	10	6.8
**Urology**	8	5.5
**Psychiatry**	6	4.1
**Orthopedics**	4	2.7
**General Surgery**	4	2.7
**Ophthalmology**	4	2.7
**Dental/Maxillofacial surgery**	4	2.7
**Anesthesia**	6	4.1
**Cardiothoracic surgery**	4	2.7
**Oncology**	2	1.4
**Plastic surgery**	2	1.4
**Radiology**	2	1.4
**Dermatology**	2	1.4
**Years of Practice**		
**More than 20 years**	94	64.4
**10–20 years**	36	24.7
**5–10 years**	16	11
**Less than 5 years**	-	-

ENT – ear, nose and throat.

During the COVID-19 pandemic, 62% of respondents felt that there was a reduction greater than 50% in outpatient visits and the majority (63%) of them felt that the situation would only improve in one to two years.

Almost all (91.8%) of the respondents were already using an electronic patient record management system at the survey time. Unfortunately, only 22% of respondents reported using telemedicine for consultations during the COVID-19 pandemic. In regards to what percentage of patients would benefit from telemedicine, the majority (42.5%) agreed that less than 10% would benefit from it, 31.5% responded between 10–30%, 15% responded between 30 to 50%, and 9.6% responded above 50%.

When questioned about the future of telemedicine, 34.2% of doctors found telemedicine useful in situations similar to COVID-19, 42.5% of doctors found it useful regardless of the occasion and thought it should be integrated as a normal part of clinical practice and 23.3% of doctors felt that telemedicine was not relevant to their practice. The majority (67.1%) agreed that telemedicine was best suited for follow-up cases.

Awareness of the organizational readiness in implementing telemedicine is essential. Unfortunately, 39.7% had no knowledge of the drug delivery services available at their respective hospital.

In 1997, the Malaysian government implemented the Telemedicine Act as a guideline and proposed a protocol for clinicians to practice telemedicine. Unfortunately, 43.8% had no knowledge of the act and this proved to be a significant barrier in the implementation of telemedicine in the Malaysian healthcare system.

Table 2 shows the comparison of responses between male and female respondents. 80% of female respondents answered that at least 10% or more of their patients would benefit from telemedicine compared to 45.8% of male respondents (P=0.03). Other questions about the impact of COVID-19 on healthcare economics, intention and willingness to use telemedicine, knowledge and awareness of telemedicine, and organization readiness showed no statistically significant differences between male and female responses.

**Table 2.** Comparison of questionnaire responses between male and female medical doctors.

**Percentage % (N)**				
**Questions**	**Answers**	**Male (N=96)**	**Female (N=50)**	**P-Value**
**Impact of COVID-19 on Healthcare Economics**				
**Estimated reduction in outpatient visit during COVID-19**	10–30%	4.17 (4)	20 (10)	0.0595
	30–50%	33.33 (32)	12 (6)	
	More than 50%	60.42 (58)	64 (32)	
	Not Affected	2.08 (2)	4 (2)	
**When will COVID-19 improve in Malaysia?**	Next 6 months	31.25 (30)	36 (18)	0.4315
	1–2 years	62.50 (60)	64 (32)	
	Never	6.25 (6)	0	
**Intention and willingness to use telemedicine**				
**Are you currently practicing Telemedicine?**	Yes, before the COVID-19 outbreak	8.33 (8)	12 (6)	0.2808
	Yes, after the COVID-19 outbreak	8.33 (8)	20 (10)	
	No	83.33 (80)	68 (34)	
**What is the future of Telemedicine in Malaysia?**	Only useful in situation similar to COVID-19	33.33 (32)	36 (18)	0.0643
	Useful and should be part of my daily practice	35.42 (34)	36 (18)	
	Not relevant to my practice	31.25 (30)	28 (14)	
**Percent of patients that would benefit from Telemedicine**	Less than 10 %	54.17 (52)	20 (10)	0.0293
	10–30%	22.92 (22)	48 (24)	
	30–50%	14.58 (14)	16 (8)	
	More than 50%	8.33 (8)	16 (8)	
**Knowledge and awareness of Telemedicine**				
**Which group of patients is suitable for Telemedicine?**	New cases	2.08 (2)	4 (2)	0.6245
	Follow-up cases	70.83 (68)	60 (30)	
	Both groups	27.08 (26)	36 (18)	
**Where did you learn about Telemedicine?**	Not heard of it	4.17 (4)	4 (2)	0.5069
	News or social media	75.00 (72)	60 (30)	
	Hospital Management	18.75 (18)	28 (14)	
	Friends and family	0	4 (2)	
	Government /Ministry of Health	2.08 (2)	4 (2)	
**Organization Readiness**				
**Do you use IT solutions for patient record management?**	Yes	89.58 (86)	96 (48)	0.6571
	No	10.42 (10)	4 (2)	
**Does your practice provide delivery of prescriptions?**	Yes	35.42 (34)	48 (24)	0.3236
	No	64.58 (62)	52 (26)	
**1997 Act of Telemedicine. Should it be reviewed?**	Yes	52.08 (50)	60 (30)	0.6582
	No	2.08 (2)	0	
	I have no knowledge of the Act	45.83 (44)	40 (20)	

Table 3 shows the comparison of responses between surgical specialties and non-surgical specialties. The reduction of outpatient volume during COVID-19 was observed equally in both surgical (61.8%) and non-surgical practices (61.5%), with the vast majority (61.6%) of respondents seeing 50% or fewer than their typical volume of patients. Significant differences (P=0.03) were recorded regarding the intention and willingness to use telemedicine, as 51.3% of non-surgeons agreed that telemedicine was useful and should be part of their daily practice compared to 32.4% in the group of surgeons. In the group of surgical specialties, 50% of doctors answered telemedicine was only useful in situations similar to COVID-19 compared to 20.5% in the non-surgical group. There were significant differences (P=0.01) in terms of knowledge and awareness of the 1997 Malaysian act of telemedicine, with 59% of doctors from non-surgical specialties reporting no knowledge on the existence of the act compared to 26.5% of surgeons.

**Table 3.** Comparison of questionnaire responses between surgeons and non-surgeons.

**PERCENTAGE % (N)**				
**Questions**	**Answers**	**Non-surgical specialties (N=78)**	**Surgical specialties (N= 68)**	**P-Value**
**Impact of COVID-19 on Healthcare Economics**				
**Estimated reduction in outpatient visits during COVID-19**	10–30%	10.26 (8)	8.82 (6)	0.9968
	30–50%	25.64 (20)	26.47 (18)	
	More than 50%	61.54 (48)	61.76 (42)	
	Not Affected	2.56 (2)	2.94 (2)	
**When will COVID-19 improve in Malaysia?**	Next 6 months	38.46 (30)	26.47 (18)	0.4527
	1–2 years	56.41 (44)	70.59 (48)	
	Never	5.13 (4)	2.94 (2)	
**Intention and willingness to use telemedicine**				
**Are you currently practicing Telemedicine?**	Yes, before the COVID-19 outbreak	10.26 (8)	8.82 (6)	0.8387
	Yes, after the COVID-19 outbreak	10.26 (8)	14.71 (10)	
	No	79.49 (62)	76.47 (52)	
**What is the future of Telemedicine in Malaysia?**	Only useful in situation similar to COVID-19	20.51 (16)	50.00 (34)	0.03
	Useful and should be part of my daily practice	51.28 (40)	32.35 (22)	
	Not relevant to my practice	28.21 (22)	17.65 (12)	
**Percent of patients that would benefit from Telemedicine**	Less than 10 %	38.46 (30)	47.06 (32)	0.1144
	10–30%	41.03 (32)	23.53 (16)	
	30–50%	7.69 (6)	23.53 (16)	
	More than 50%	12.82 (10)	5.88 (4)	
**Knowledge and awareness of Telemedicine**				
**Which group of patients is suitable for Telemedicine?**	New cases	5.13 (4)	0	0.3966
	Follow-up cases	66.67 (52)	67.65 (46)	
	Both groups	28.21 (22)	32.35 (22)	
**Where did you learn about Telemedicine?**	Not heard of it	7.69 (6)	0	0.1266
	News or social media	58.97 (46)	82.35 (56)	
	Hospital Management	25.64 (20)	17.65 (12)	
	Friends and family	2.56 (2)	0	
	Government /Ministry of Health	5.13 (4)	0	
**Organization Readiness**				
**Do you use IT solutions for patient record management?**	Yes	92.31 (72)	91.18 (62)	1(0.5948)
	No	7.69 (6)	8.82 (6)	
**Does your practice provide delivery of prescriptions?**	Yes	48.72 (38)	9.41 (20)	0.1018
	No	51.28 (40)	70.59 (48)	
**1997 Act of Telemedicine. Should it be reviewed?**	Yes	41.03 (32)	70.59 (48)	0.0148
	No	0	2.94 (2)	
	I have no knowledge of the Act	58.97(46)	26.47 (18)	

Table 4 shows the comparison of responses between different medical disciplines. There were significant differences (P= 0.01) in the reduction of outpatient volume between different disciplines. Internal medicine physicians (89.9%), emergency physicians (66.7%), pediatricians (66.7%) and Ear, Nose and Throat (ENT) surgeons (64.3%) reported a reduction greater than 50% in the outpatient volume. However, doctors in obstetrics and gynecology (60%) observed a 10 to 20% reduction in the outpatient volume.

**Table 4.** Comparison of questionnaire responses between doctors from various disciplines.

**PERCENTAGE % (N)**										
**Questions**	**Answers**	**ENT (n=28)**	**Emergency Medicine (n=18)**	**Internal Medicine (n=18)**	**Family Medicine (n=12)**	**Pediatrics (n=12)**	**O&G (n=10)**	Others* (n=48)	**Total (n=146)**	**P-Value**
**Impact of COVID-19 on Healthcare Economics**										
**Estimated reduction in outpatient visit during COVID-19**	10-30%	-	-	-	33.33(4)	16.7(2)	60(6)	4.2(2)	9.6(14)	0.0122
	30-50%	35.7(10)	33.3(6)	11.1(2)	16.67(2)	16.7(2)	-	33.3(16)	26.0(38)	
	More than 50%	64.3(18)	66.7(12)	88.9(16)	50.00(6)	66.7(8)	20(2)	58.3(28)	61.6(90)	
	Not Affected	-	-	-	-	-	20(2)	4.2(2)	2.7(4)	
**When will COVID-19 improve in Malaysia?**	Next 6 months	35.7(10)	33.3(6)	44.4(8)	50.00(6)	50(6)	20(2)	20.8(10)	32.9(48)	0.7946
	1-2 years	64.3(18)	55.6(10)	44.4(8)	50.00(6)	50(6)	80(8)	75(36)	63.0(92)	
	Never	-	11.1(2)	11.1(2)	-	-	-	4.2(2)	4.1(6)	
**Intention and willingness to use telemedicine**										
**Are you currently practicing Telemedicine?**	Yes, before the COVID-19 outbreak	7.1(2)	-	-	-	33.3(4)	20(2)	12.5(6)	9.6(14)	0.6104
	Yes, after the COVID-19 outbreak	7.1(2)	22.2(4)	11.1(2)	16.67(2)	-	-	16.7(8)	12.3(18)	
	No	85.7(12)	77.8(14)	88.9(16)	83.33(10)	66.7(8)	80(8)	70.8(34)	78.1(114)	
**What is the future of Telemedicine in Malaysia?**	Only useful in situation similar to COVID-19	50(14)	-	22.2(4)	16.67(2)	50(6)	40(4)	41.7(20)	34.2(50)	0.0607
	Useful and should be part of my daily practice	42.9(12)	88.9(16)	55.56(10)	33.33(4)	50(6)	20(2)	25(12)	42.5(62)	
	Not relevant to my practice	7.1(2)	11.1(2)	22.2(2)	50.00(6)	-	40(4)	33.3(16)	23.3(34)	
**Percent of patients that would benefit from Telemedicine**	Less than 10 %	50(14)	44.4(8)	33.3(6)	33.33(4)	-	40(4)	54.2(26)	42.5(62)	0.4477
	10-30%	21.4(6)	22.2(4)	55.6(10)	33.33(4)	83.3(10)	20(2)	20.8(10)	31.5(46)	
	30-50%	14.3(4)	11.1(2)	11.1(2)	16.67(2)	-	40(4)	20.8(10)	16.5(24)	
	More than 50%	14.3(4)	22.2(4)	-	16.67(2)	16.7(2)	-	4.2(2)	9.59(14)	
**Knowledge and awareness of Telemedicine**										
**Which group of patients is suitable for Telemedicine?**	New cases	-	11.1(2)	-	-	-	-	4.2(2)	2.7(4)	0.8706
	Follow-up cases	71.4(20)	66.7(12)	55.56(10)	83.33(10)	83.3(10)	80(8)	58.3(28)	67.1(98)	
	Both groups	28.6(8)	22.2(4)	44.44(8)	16.67(2)	16.7(2)	20(2)	37.5(18)	30.1(44)	
**Where did you learn about Telemedicine?**	Not heard of it	-	22.2(4)	11,1(2)	-	-	-	-	4.1(6)	0.1389
	News or social media	85.7(24)	77.8(14)	44.4(8)	66.67(8)	50(6)	60(6)	75.00(36)	69.9(102)	
	Hospital Management	14.3(4)	-	33.3(6)	33.33(4)	33.3(4)	40(4)	20.83(10)	21.9(32)	
	Friends and family	-	-	-	-	16.7(2)	-	-	1.4(2)	
	Government/Ministry of Health	-	-	11.1(2)	-	-	-	4.17(2)	2.7(4)	
**Organization Readiness**										
**Do you use IT solutions for patient record management?**	Yes	85.7(24)	100(18)	88.9(16)	66.67(8)	100(12)	80(8)	100(48)	91.8(134)	0.116
	No	14.3(4)	-	11.1(2)	33.33(4)	-	20(2)	-	8.2(12)	
**Does your practice provide delivery of prescriptions?**	Yes	35.7(10)	44.4(8)	55.6(10)	33.33(4)	33.3(4)	20(2)	41.7(20)	39.7(58)	0.903
	No	64.3(18)	55.6(10)	44.4(8)	66.67(8)	66.7(8)	80(8)	58.3(28)	60.3(88)	
**1997 Act of Telemedicine. Should It be reviewed?**	Yes	71.4(20)	44.4(8)	44.4(8)	50.00(6)	50(6)	60(6)	54.2(23)	54.8(80)	0.8111
	No	7.1(2)	-	-	-	-	-	-	1.4(2)	
	I have no knowledge of the Act	21.4(6)	55.6(10)	55.6(10)	50.00(6)	50(6)	40(4)	45.8(22)	43.8(64)	

O&G – obstetrics and gynecology. * Includes responses from the following disciplines: urology, psychiatry, orthopedics, general surgery, ophthalmology, dental/maxillofacial surgery, anesthesia, cardiothoracic surgery, oncology, plastic surgery, radiology and dermatology.

Table 5 shows the comparison of responses between those that adopted telemedicine before and after the COVID-19 pandemic. There were no statistical differences noted in the responses between the two groups. However, doctors that adopted telemedicine after COVID-19 reported a higher acceptance of the technology (88.9%). Another notable difference between the two groups was related to the types of patients suitable for the practice of telemedicine. 85.7% of those that adopted telemedicine earlier felt that the technology is best suited for follow-up patients only. On the contrary, the majority of those who adopted telemedicine after COVID-19 reported that both new and follow-up patients are suitable for telemedicine (55.6%).

**Table 5.** Comparison of questionnaire responses between doctors that adopted telemedicine before and after the COVID-19 pandemic.

****PERCENTAGE % (N)****				
**Questions**	**Answers**	**Practicing Telemedicine before COVID-19 (N=14)**	**Practicing telemedicine after COVID-19 (N=18)**	**P-Value**
**Impact of Covid on Healthcare Economics**				
**Estimated reduction in outpatient visit during COVID-19**	10-30%	14.3 (2)	-	0.1913
	30-50%	14.3 (2)	-	
	More than 50%	57.1 (8)	100(18)	
	Not Affected	14.3 (2)	-	
**When will COVID-19 improve in Malaysia?**	Next 6 months	-	55.6(10)	0.9494
	1-2 years	42.9 (6)	44.4(8)	
	Never	57.1 (8)	-	
**Intention and willingness to use telemedicine**				
**What is the future of Telemedicine in Malaysia?**	Only useful in situation similar to COVID-19	42.9 (6)	-	0.0756
	Useful and should be part of my daily practice	57.1 (8)	88.9 (16)	
	Not relevant to my practice	-	11.1 (2)	
**Percent of patients that would benefit from Telemedicine**	Less than 10 %	28.6 (4)	33.3 (6)	0.5467
	10-30%	57.1 (8)	33.3 (6)	
	30-50%	-	22.2 (4)	
	More than 50%	14.3(2)	11.1 (2)	
**Knowledge and awareness of Telemedicine**				
**Which group of patients is suitable for Telemedicine?**	New cases	-	11.1 (2)	0.1061
	Follow-up cases	85.7 (12)	33.3 (6)	
	Both groups	14.3 (2)	55.6 (10)	
**Where did you learn about Telemedicine?**	Not heard of it	-	-	0.8385
	News or social media	71.4 (10)	66.7 (12)	
	Hospital Management	28.6 (4)	33.3 (6)	
	Friends and family	-	-	
	Government /Ministry of Health	-	-	
Organization Readiness				
**Do you use IT solutions for patient record management?**	Yes	100 (14)	100 (18)	-
	No	-	-	
**Does your practice provide delivery of prescriptions?**	Yes	57.1 (8)	33.3 (6)	0.3409
	No	42.9 (6)	66.7 (12)	
**1997 Act of Telemedicine. Should It be reviewed?**	Yes	42.9 (6)	44.4 (8)	0.9494
	No	-	-	
	I have no knowledge of the Act	57.1 (8)	55.6 (10)	

**Figure 1.**
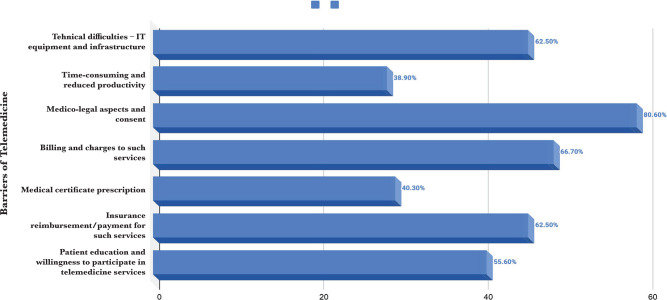
Barriers against the use of telemedicine (percentages).

A variety of different reasons were cited by respondents as barriers against the use of telemedicine, including medico-legal aspect and consent (80.6%), billing and charges for such services (66.7%), insurance reimbursement/payment for such services (62.5%), technical difficulties including the setup and availability of technology (62.5%), patients willingness to adopt telemedicine (55.6%), time consumption and reduction in productivity (38.9%) and prescription of a medical certificate (40.3%) (Figure 1).

## Discussion

### Interpretation of the main findings

In this study, 34.2% of doctors agreed telemedicine was useful in situations similar to the pandemic of COVID-19, and the majority felt that it should be integrated as a normal part of clinical practice (42.5%). There was still a minority (23%) of doctors who felt that telemedicine was not relevant to their practice. These findings were similar to the study conducted by Ibrahim *et al.* in 2010, where almost 80% of doctors were in favor of the idea of remote communication with their patients [16].

Despite the majority (62%) of the doctors experiencing a reduction greater than 50% in outpatient visits during the COVID-19 pandemic, there was no significant increase in doctors practicing telemedicine when compared to before the pandemic. Only a handful of doctors (14%) practiced telemedicine before the COVID-19 pandemic, and it increased to 12% after the pandemic began. This was partly due to the negative perception of Malaysian doctors, as the majority (74%) felt that telemedicine would only benefit up to 30% of their patients. The majority of the surgeons (67.7%) felt that telemedicine was only essential during a pandemic such as COVID-19. This corresponds to a previous study in Malaysia that shows 67.5% of clinicians were unwilling to accept a reduction in face-to-face consultations [16], probably due to the poor knowledge of doctors in practicing telemedicine and the unpreparedness of the organization of the respective hospital in implementing the system.

Age of the doctors and seniority in practice may be another explanation for the poor acceptance of telemedicine. In this study, the majority of doctors were senior clinicians (64%) who have practiced medicine for more than 20 years. A similar study conducted by Gaggioli *et al.* in Milan found that senior male doctors were more reluctant to adopt telemedicine for a variety of reasons [17]. This could also explain our findings in a subgroup analysis where female doctors (80% vs. 45.8%) were more optimistic than their male counterparts. In our study, most female respondents were in the younger age group, with 48% practicing for less than 20 years. Our findings differed from previous studies, which stated that male doctors were more likely to adopt telemedicine [17, 18].

This study also showed that physicians (51.3%) are more willing to adopt telemedicine than doctors from surgical specialties (32.4%). Surgical subspecialties are usually a barrier for telemedicine due to their procedural component [19]. Ophthalmologists, for example, may find that telemedicine is limiting their patients’ eye physical examination with an ophthalmoscope or slit lamp that would be required to reach a diagnosis or progress of the disease. Unfortunately, this is not valid in all surgical specialties. Telemedicine is still beneficial, especially during the preoperative and postoperative assessment, thus minimizing the attendance of patients to the hospital, which has a significant impact on the cost of patient care [20]. For example, there was increasing concern about the safety of various endoscopic and laparoscopic procedures during the COVID-19 pandemic in general surgery and colorectal surgery. There appears to be a potential of virus spread with the utilization of laparoscopy, mainly due to aerosolization of bodily fluids and vapor formed by heat-generating cautery devices [21, 22]. Various studies have been published to encourage a conservative approach in managing such cases, with endoscopy and proctological procedures performed selectively [23]. Telemedicine has been suggested as a tool for consultation and screening; only those who are deemed not deferrable are attended physically in the hospital [23].

Besides the role in patient teleconsultation, telemedicine is also playing an increasingly important role as a tool to obtain clinical guidelines and communications among team members. A survey done by Benítez *et al.* concluded that social media and video conferences were the most popular options utilized during the COVID-19 pandemic for such purpose while maintaining social distancing [24].

Another notable mention is the role of telemedicine in the field of psychiatry and mental health. Among the various impacts of COVID-19, fear of infection and uncertainty about the disease can precipitate various psychiatric disorders; those with predisposing mental illness may experience more worry, anxiety, and suicidal ideation and develop other mental illnesses in comparison to healthy controls [25]. Telepsychiatry and smartphone-based cognitive therapy has been demonstrated to be an effective option where psychiatric patients tend to overestimate the risk of contracting COVID-19 [26].

There were multiple concerns regarding the implementation of telemedicine and why it would benefit a minority of patients in this study. Primarily, the doctors were concerned about the medico-legal, security and privacy implications of telemedicine (80.6%). Medico-legally, doctors are at risk, as there is a lack of rules, legislation, and updated protocols for telemedicine, unlike its more traditional face-to-face counterpart [27]. For example, when a misdiagnosis occurs during a virtual consultation, the fault may lie with the technology, doctor, or patient. Therefore, the legal processes to overcome it are far more complicated than a similar error occurring during a face-to-face consultation where the law is standardized and universal.

The Malaysian Medical Council (MMC) advisory on telemedicine during the COVID-19 pandemic states that medical practitioners must possess adequate training, require valid informed consent, confirm the identity of patients, patient approval on other parties present and make sure that the technology used complies with legal requirements regarding privacy and security. However, the biggest drawback to the advisory was the requirement for doctors to reserve the practice of telemedicine solely for patients under their care. This may be a limitation of telemedicine in the COVID-19 era as it prevents doctors from conducting telemedicine consults on new patients who have no access to the hospital during the pandemic [28]. The Malaysian Code of Professional Conduct states that physical examination is mandatory for a patient consult. However, the advisory on telemedicine has given a leeway for non-physical telemedicine applied during the pandemic and not after.

The second concern was reimbursement or bill payments for telemedicine services. Questions arise if consultation fees via telemedicine should be based on current guidelines of face-face consultations or if they should be reduced as no physical examination is performed. However, this does not resolve the fact that the doctor is still consulting, interpreting results, and providing professional advice during telemedicine via audio-only phone calls, video calls, text chat, or e-mails.

Currently, in Malaysia, online health providers cite a fee of about 20 Ringgit Malaysia (RM) (4.70 USD) for a consult with a general practitioner and 40 RM (9.40 USD) for a consult with a specialist [29]. These charges are much lower than a face-to-face consultation at private clinics or hospitals. Hence, they may not be financially viable given the substantial initial investment of telemedicine. This could negatively affect the income of clinicians if traditional fee-for-service payment methods continue to be followed.

The third concern in implementing telemedicine was the technical difficulties. Doctors felt that the current environment lacked the adequate infrastructure for telemedicine (62.5%). This included Internet bandwidth, network issues, proper video conferencing applications, and reliable data storage. This corresponded to a study in Saudi Arabia where only 33.3% of clinicians were actually implementing telemedicine in hospitals that adopted it. Reasons cited were technical issues, insufficient training for doctors, and poor response from patients who preferred face-to-face consultations [18]. In terms of security and privacy issues, doctors were skeptical that the current systems available were safe and secured (i.e., not easily hacked or spied on). During the COVID-19 pandemic, there have been issues regarding patient privacy intrusions through the use of tools such as the Zoom Communication Inc. application for video conferencing [30]. These tools are simple, inexpensive, and easy to use by healthcare providers and patients alike. However, they lead to issues such as hacking intrusions and non-secure storage of video data. In the USA, doctors risk facing lawsuits or state actions for patient privacy violations despite acting in “good faith” to provide telemedicine services during the COVID-19 pandemic [30]. Therefore the concern for data privacy and security is critical.

The other concerns include patient education and willingness to participate (55.6%). This agreed with a recent study conducted in an urban city in Malaysia where less than half of its 4504 respondents found that the role of the Internet or a mobile health application was beneficial to them [31]. The authors indicated that this was due to most respondents having limited knowledge regarding the use of online health applications and the cost of embracing such technology [31]. Even in the neighboring country of Singapore, an urban center with one of the highest penetration of information technology globally, a recent study found that only 52.5% of their population was willing to use telemedicine [32]. Deterrents to telemedicine included age, ethnicity, patients’ beliefs, cost and privacy [32].

Finally, doctors were also concerned over the legality of online medical certificate (MC) prescription (40.4%). This concern stems from an age-old tradition where MCs were required to be stamped with a doctor’s Medical Council stamp in order for employers to verify its validity and authenticity [33]. In our study, the majority of doctors were comfortable with online MC prescriptions.

### Limitations

The response rate of 41.7% was considered low, but it would be understandable as many are struggling to cope with the unprecedented changes in their daily practice. This study had participants mainly from a private healthcare system where most doctors were senior consultants, with many having above 20 years of experience. It is possible that the sampling method may have led to a self-selecting bias, in which doctors who were particularly willing and able to practice telemedicine did not participate. This study only targets a niche population in urban private institutions covering four neighboring states in West Malaysia. An inclusive study should include the states of East Malaysia (i.e., Sabah and Sarawak) with a larger rural community and doctors from public hospitals where healthcare accessibility and patient congestion represent an issue.

This study had participants mainly from disciplines that require an in-person physical examination and may be underrepresented by those from disciplines where telemedicine is more suited.

### Recommendations

According to Bashshur *et al.*, the success of telemedicine rests on the three pillars of care: improved access, enhanced quality, and cost containment [34]. Therefore, firstly, the adoption of telemedicine has to be a government initiative. This will allow for standardization of guidelines and wider access to the public [35]. The benefits of a centralized system with government support have been shown briefly during this COVID-19 pandemic through the initiation of online tracking applications (i.e., MySejahtera, Selangkah) [36]. Although in their infancy, these applications have been able to rapidly attain widespread use throughout the country through extensive media coverage and issuance of incentives for downloading this application [36]. The benefits of these government-supported telemedicine initiatives have seen similar responses in other countries such as China, Taiwan, Korea, and Singapore [37].

Secondly, telemedicine needs to be financially viable for both healthcare providers and the public. Policy-makers and insurance providers should provide clarity on reimbursements on the various telemedicine encounters and pivot away from the traditional payment models [27]. This has been observed in multiple countries (e.g., United Kingdom, Germany, Sweden, France, Australia) since the start of the COVID-19 pandemic [38, 39]. In those countries, government and insurance companies have moved with an uncharacteristic speed to have various teleconsultations covered.

Thirdly, education initiatives would be needed for both clinicians and the public. As for clinicians, they should be informed about developing legislation and regulatory developments in telemedicine through conferences and seminars [24]. For the public, there needs to be more dissemination of information from healthcare providers, schools, and professional associations regarding the healthcare resources and support available through telemedicine.

Lastly, this study provides an insight into the perception of telemedicine amongst medical practitioners in the region prior to and during COVID-19. The recommendations will serve as an implementation guide for countries whose regions had limited use of telemedicine prior to the COVID-19 outbreak.

## Conclusion

This study has shown that female doctors and physicians have a more positive outlook on telemedicine and are willing to participate when compared to male doctors and doctors from surgical specialties. However, regardless of gender, specialty, seniority, or loss of income, the majority of doctors perceived that telemedicine would only benefit up to 30% of their patients. The main barriers were medico-legal issues due to lack of proper legislation, reimbursement of services, technical difficulties, patient education and willingness to participate. The recommendations to improve the adoption of telemedicine include government engagement, fee standardization, and education initiatives. Therefore, although the COVID-19 pandemic appeared to improve the perception of telemedicine among clinicians and their willingness to adopt it, in reality, there are significant barriers that need to be resolved, and few doctors would truly implement telemedicine in Malaysia.

## Acknowledgments

### Ethical approval

The approval for this study was obtained from the Ethics Committee of the KPJ Healthcare University College, Negeri Sembilan, Malaysia (approval ID: KPJUC/RMC/EC/2020/283).

### Consent to participate

The participants entered the study voluntarily and their confidentiality was kept.

### Conflict of interest

The authors declare that there is no conflict of interest.
